# Supplementary material for: Density Functional Theory Calculations and Molecular Docking Analyses of Flavonoids for Their Possible Application against the Acetylcholinesterase and Triose-Phosphate Isomerase Proteins of Rhipicephalus microplus
Source: Molecules. 2023 Apr 20;28(8):3606. doi: 10.3390/molecules28083606 (PMC10145301; doi:10.3390/molecules28083606)

**Supplementary Figure S1.** Two-dimensional (2D) structures of flavonoid compounds.

**Supplementary Figure S2.** The molecular docking of RmAChE1 showed reacting sites with quercetagetin-7-O-(6-O-caffeoyl-β-D-glucopyranoside) in (A) 3D and (B) 2D pictures.


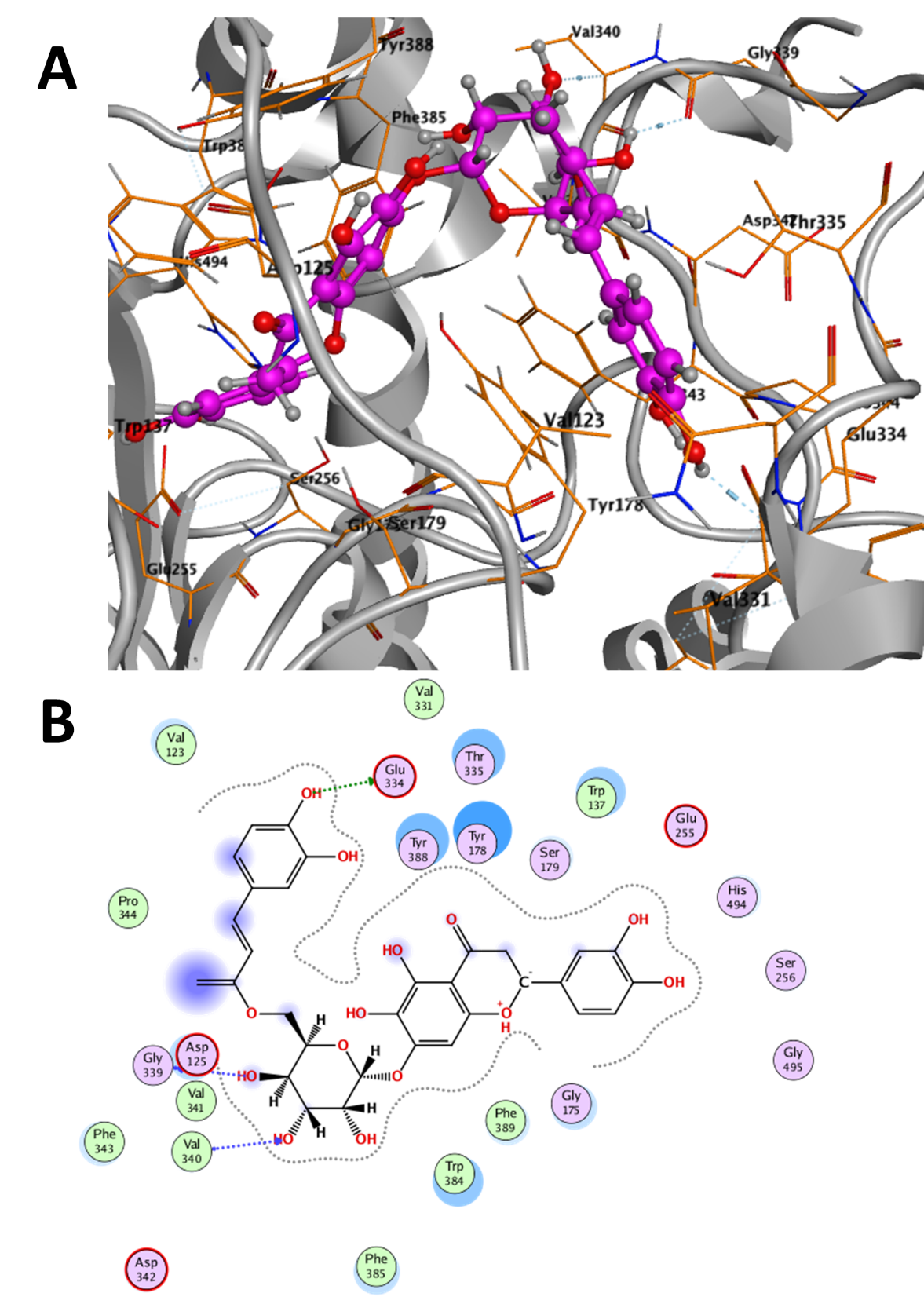


**Supplementary Figure S3.** The molecular docking of RmAChE1 showed reacting sites with quercetagetin-7-O-(6-O-p-coumaroyl-β-glucopyranoside) in (A) 3D and (B) 2D pictures.


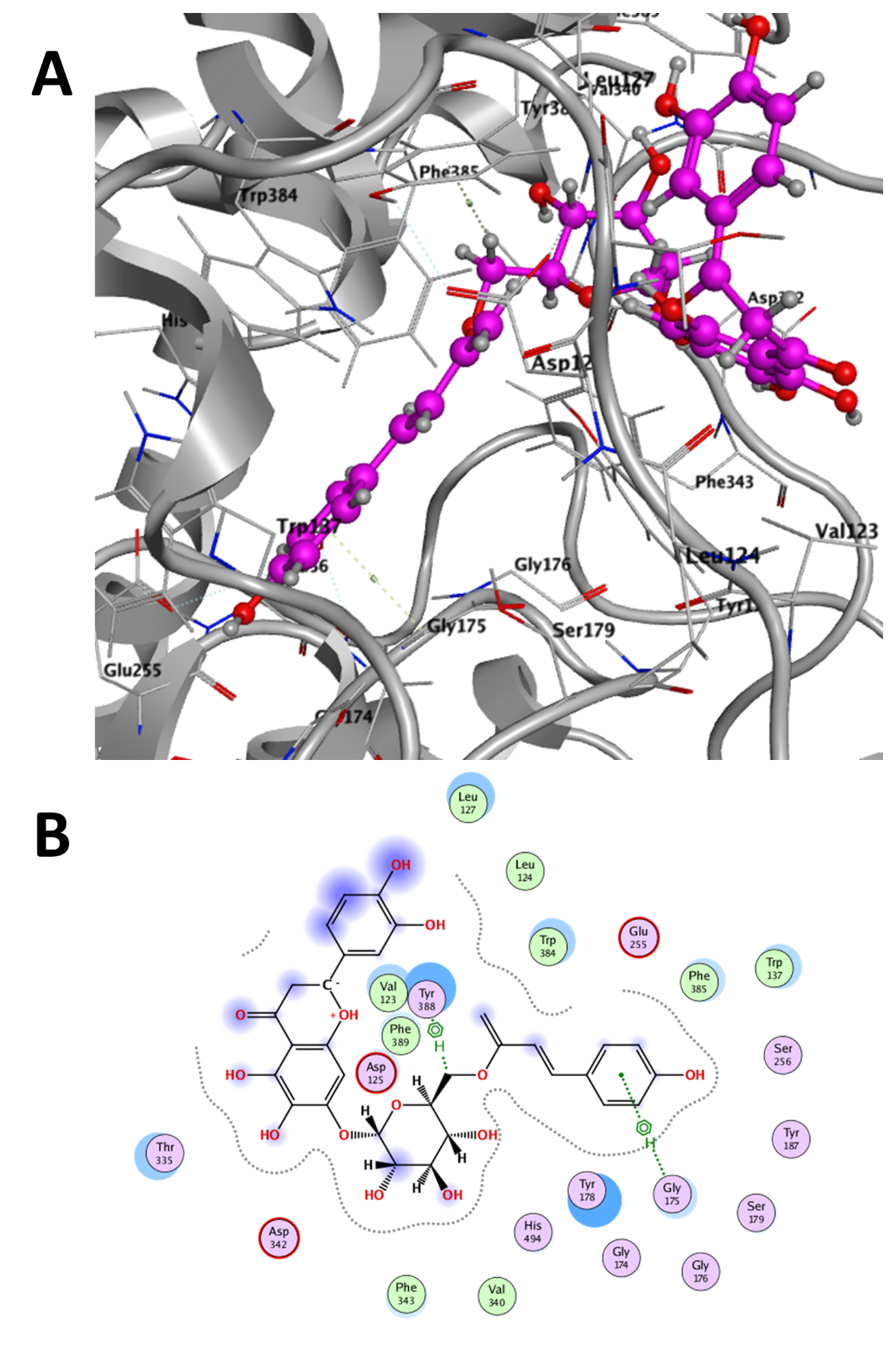


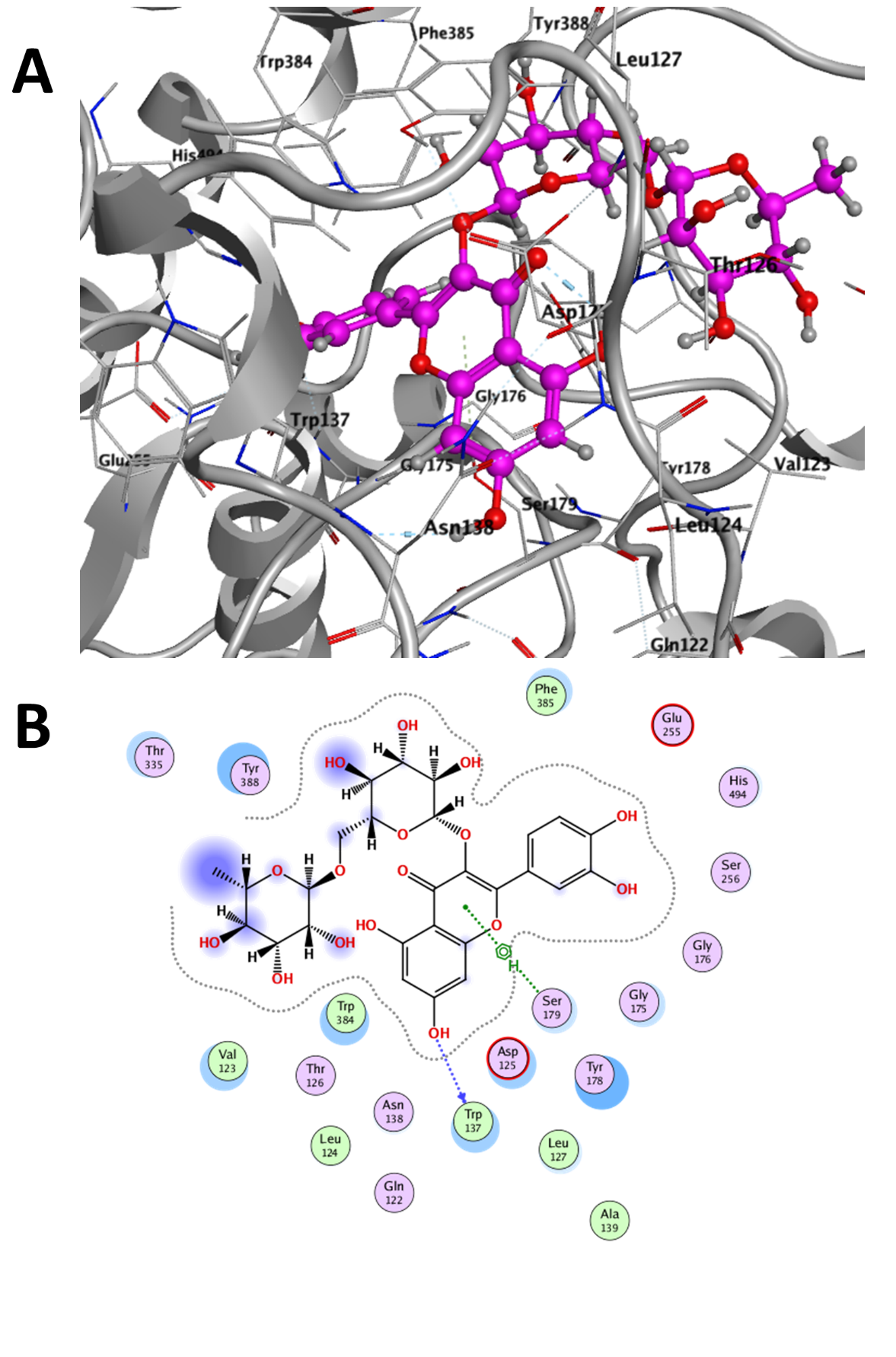
**Supplementary Figure S4.** The molecular docking of RmAChE1 showed reacting sites with rutin in (A) 3D and (B) 2D pictures.

**Supplementary Figure S5.** The molecular docking of RmAChE1 showed reacting sites with kaempferol 3-neohesperidoside in (A) 3D and (B) 2D pictures.
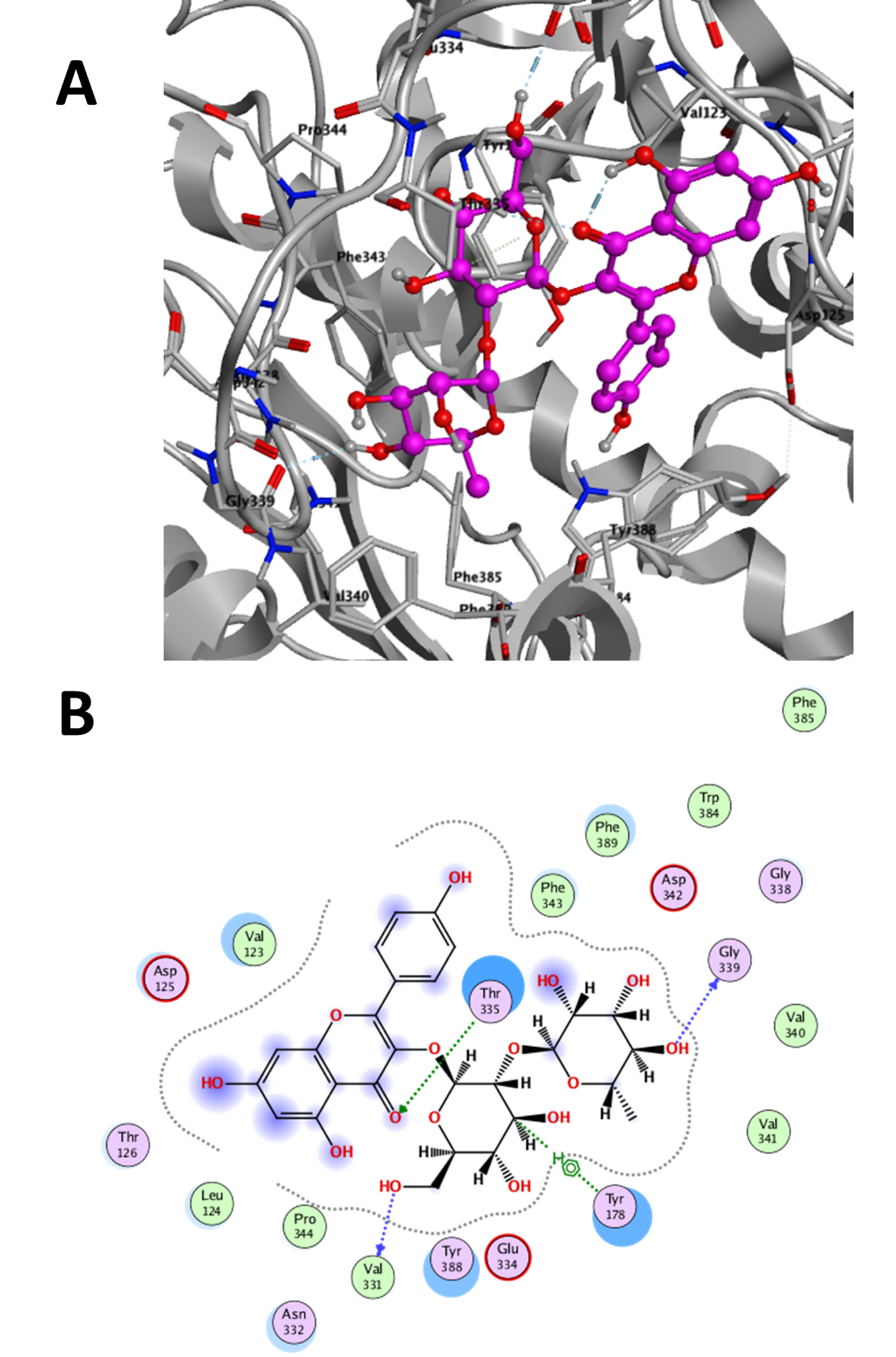

Supplement: Supplementary file 1 [file molecules-28-03606-s001.zip › molecules-2115310-supplementary.docx]
